# Is Wildfire Suppression in Giant Sequoia Groves a Problem, a Solution, or Neither?

**DOI:** 10.1002/ece3.73286

**Published:** 2026-03-16

**Authors:** Chad T. Hanson

**Affiliations:** ^1^ Earth Island Institute Berkeley California USA

**Keywords:** fire suppression, giant sequoia forests, high‐severity wildfire, prescribed fire

## Abstract

In 1910, the U.S. federal government began an official policy of suppressing wildfires. Decades later it became understood that the giant sequoia, the world's most massive tree, is serotinous and depends upon fire to effectively reproduce. For a century, fire was almost completely excluded from giant sequoia groves, until a series of lightning fires over the past decade. After these fires, U.S. land managers hypothesized that the blazes had caused unprecedented levels of high‐severity fire due to a century of fire suppression and fuel accumulation. Based on this assumption, U.S. legislation is now proposed to override environmental laws to allow logging in all giant sequoia groves on federal public lands, including in Wilderness Areas and national parks, in the name of wildfire prevention. In addition, lower‐severity prescribed fires are now being implemented as a means to prevent and suppress mixed‐severity wildfires, based on the assumption that high initial post‐burn sequoia seedling densities after prescribed fire will translate to relatively high densities in later years. I investigated the effects of wildfire suppression in giant sequoia groves using GIS data of wildfire perimeters dating back to 1910, fire severity data from 2012 to present, and a government prescribed fire and sequoia regeneration dataset. I found fire of all severities since 1910 is below frequencies that occurred before fire suppression. I found no correlation between time‐since‐fire and the percentage of area burned comprised by high‐severity fire in giant sequoia groves. Further, I found no correlation between initial (1 year post‐burn) sequoia seedling densities and densities at 10 years following prescribed fire. The percentage of all plots lacking any sequoia regeneration after prescribed fire increased significantly over time. Only 23% of prescribed fire plots lacked sequoia regeneration at 1 year post‐burn, while 82% of prescribed fire plots lacked sequoia regeneration at 20 years post‐burn.

## Graphical abstract

There is much debate and speculation about whether recent large wildfires in giant sequoia groves have caused an unnatural excess of high‐severity fire, due to fire suppression and fuel accumulation. I found that fire of all severities remains in deficit since fire suppression began, relative to pre‐suppression levels, and found no correlation between time‐since‐fire (fire exclusion) and high‐severity fire percentage. I also found no correlation between initial sequoia regeneration density in lower‐severity prescribed fires, which are being used as a new form of wildfire suppression, and sequoia regeneration density at 10 years post‐fire, and 82% of prescribed fire plots were devoid of sequoia regeneration by 20 years after prescribed fire, suggesting this approach will not maintain sequoia populations.

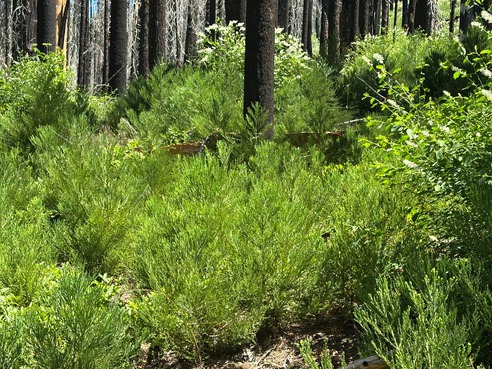

## 1 Introduction

The summer of 1910 was exceptionally hot, dry, and windy in the western United States and, unsurprisingly, it was a very large wildfire season. The largest of the 1910 wildfires spread across ~1.2 million ha of forest in the northern Rocky Mountains in just 2 days (Odion et al. 2014). In the aftermath of that fire season, U.S. federal land management agencies initiated a policy of wildfire suppression (Odion et al. 2014).

**FIGURE 1:**
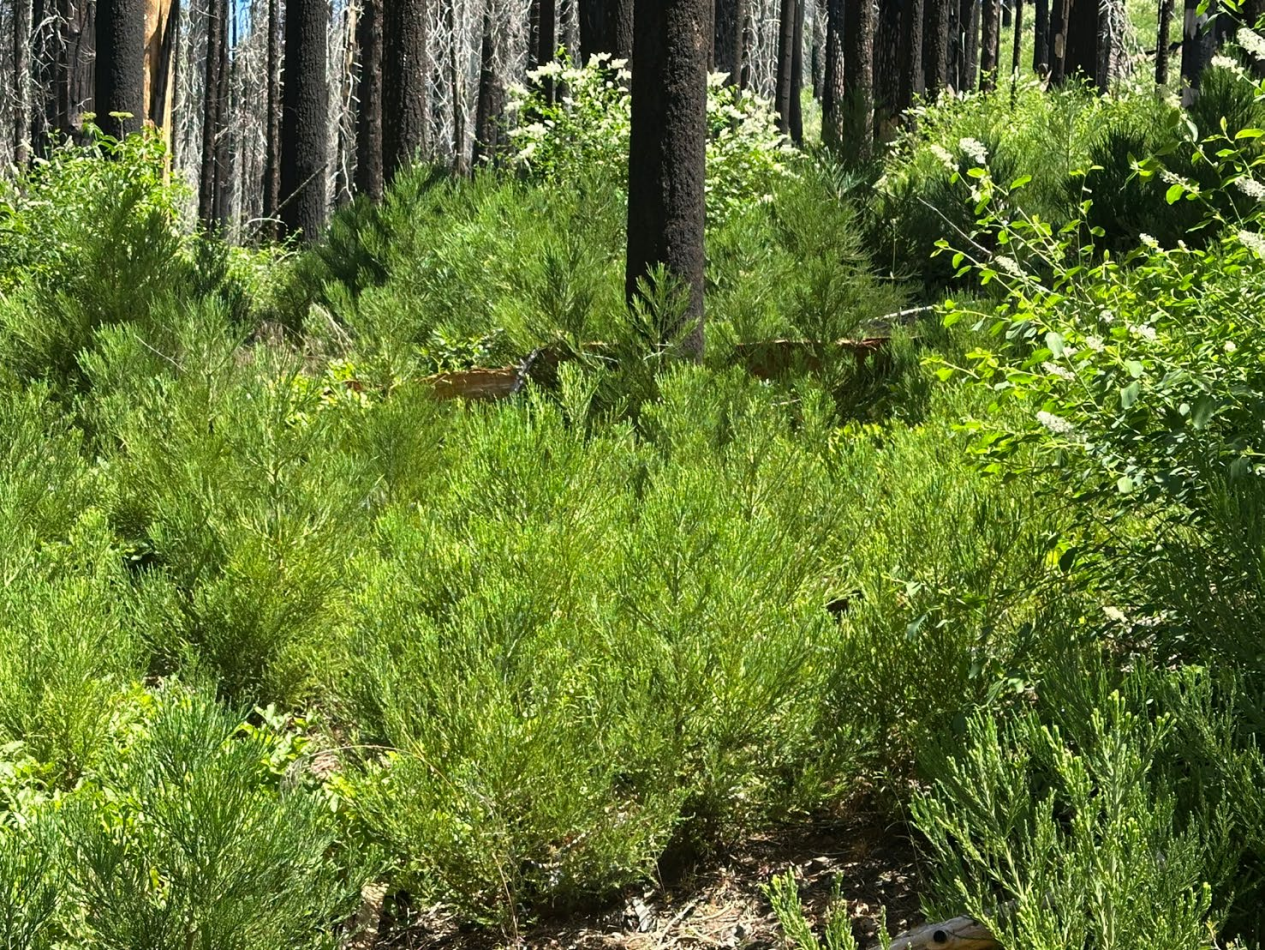
Abundant giant sequoia regeneration in a large high‐severity fire patch at 4 years after the KNP Complex fire of 2021, Redwood Mountain Grove.

In the early 20th century, this policy was thought to be the salvation of giant sequoias ( *Sequoiadendron giganteum* ), the world's most massive tree species. It was not until several decades later that some scientists began to understand that giant sequoias are serotinous (Figure 1), and depend upon fire to effectively reproduce (Harvey et al. 1980; Weatherspoon 1990; Harvey and Shellhammer 1991).

The historical fire regime in giant sequoia groves was typically dominated by low/moderate‐severity fires and scattered small high‐severity fire patches (Stephenson, Soderberg, et al. 2024; Stephenson, Caprio, et al. 2024), at short to intermediate intervals, with estimates from fire‐scar data ranging from 16 to 22 years in Swetnam et al. (2009) and 11 to 39 years in Kilgore and Taylor (1979). Fire‐free intervals of several decades up to a century or more occurred occasionally (Swetnam et al. 2009). At multi‐century time scales, historical fire regimes in giant sequoia groves also included larger high‐severity fire patches (Stephenson et al. 1991).

Despite this new understanding about giant sequoias and fire, wildfire suppression policies continued, though some limited prescribed burns were conducted in giant sequoia groves in Sequoia and Kings Canyon National Parks, or “SEKI” (Hartesveldt et al. 1975; Harvey et al. 1980; Harvey and Shellhammer 1991), and occasional smaller wildfires also occurred in SEKI. Then, beginning in 2015, a series of large wildfires occurred during hot, dry, windy conditions—fires that could not be suppressed. These were dominated by natural, lightning‐ignited fires, most of which occurred in the 2020–2021 wildfire seasons.

Shortly after the 2020–2021 fire seasons, land managers issued reports stating the assumption that the recent wildfires had caused unprecedented occurrence of high‐severity fire in giant sequoia groves, due to fire suppression policies and a century of fuel accumulation (Shive et al. 2022; Meyer et al. 2024; Soderberg et al. 2024; Stephenson, Soderberg, et al. 2024; Stephenson, Caprio, et al. 2024; Keeley and Pausas 2025). Based on this untested assumption, legislation has been proposed in the U.S. Congress that would override key environmental laws to allow logging in all giant sequoia groves on federal public lands, including in Wilderness Areas and national parks, purportedly to curb wildfires. In addition, land managers have now proposed a widespread program of lower‐severity prescribed burning to prevent and suppress further mixed‐severity wildfires (USDA 2022; Stephenson, Caprio, et al. 2024), based on the assumption that, if high sequoia seedling densities can be achieved initially in most areas through lower‐severity prescribed fire (Soderberg et al. 2024; Stephenson, Caprio, et al. 2024), this will result in relatively high regeneration densities in later years, and a stable or increasing giant sequoia population (York et al. 2013; Soderberg et al. 2024; Stephenson, Soderberg, et al. 2024; Stephenson, Caprio, et al. 2024; Keeley and Pausas 2025).

**FIGURE 2:**
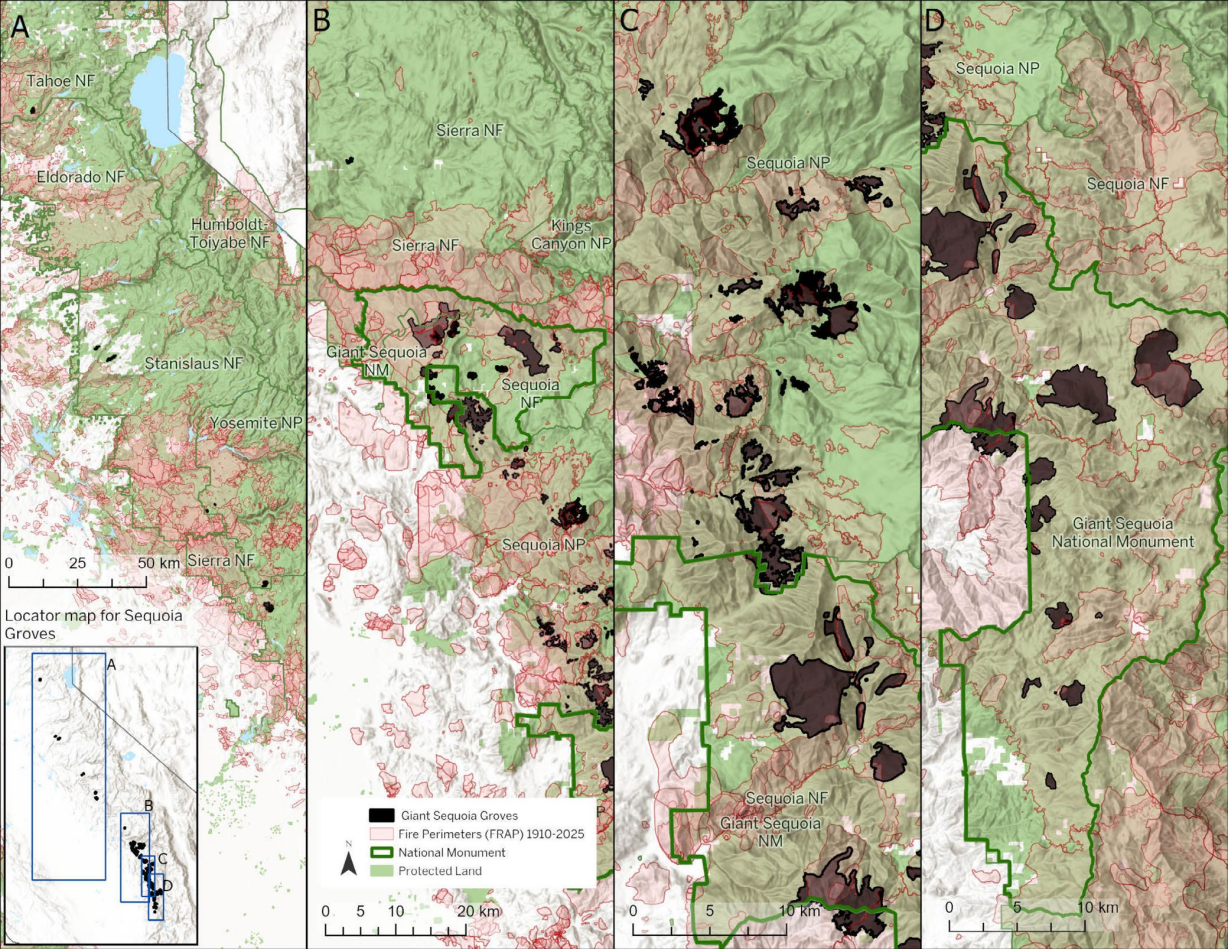
Giant Sequoia Groves and Fire Perimeters 1910–2024 in four Panels, A through D, from the northernmost groves (panel A) to the southernmost (panel D).

I investigated the effects of wildfire suppression within all extant giant sequoia groves (Figure 2). Specifically, I investigated the following: (1) the hypothesis, articulated by land managers, that fire suppression has led to an unprecedented excess of high‐severity fire in giant sequoia groves; (2) the null hypothesis that there will be no correlation between fire suppression (increasing time since the previous fire) and percent high‐severity fire; (3) the hypothesis, articulated by land managers, that initial giant sequoia seedling density after lower‐severity prescribed fire will be correlated with higher sequoia regeneration densities in later years; and (4) the null hypothesis that there will be no change in the proportion of giant sequoia groves lacking any sequoia regeneration over the course of time following lower‐severity prescribed fire.

## 2 Materials and Methods

### 2.1 Rates of High‐Severity Fire Before and After Fire Suppression

I analyzed high‐severity fire rates across all extant giant sequoia groves. I determined the post‐fire suppression (1910–2024) high‐severity fire rotation interval, defined as the time interval required for an area equal to the area of interest (the total extant area of all giant sequoia groves) to burn at high‐severity one time (Baker 2014). I used current giant sequoia grove perimeter maps provided by the National Park Service (https://nps.maps.arcgis.com/apps/instant/basic/index.html?appid=5e47ca7784734b91b4c5b004618ca87b), and I used the Rapid Assessment of Vegetation Condition (RAVG) fire severity data base (https://data.fs.usda.gov/geodata/rastergateway/ravg/index.php) to determine the hectares of high‐severity fire (76%–100% basal area mortality) in giant sequoia groves since 2012 (there were no RAVG fire data available for giant sequoia groves in years prior to 2012). RAVG fire severity data are consistently used by land managers when considering potential post‐fire management actions, so the RAVG data set is appropriate for this analysis. Then, I used the Fire and Resource Assessment Program (FRAP) data base, from the State of California, for historical fire perimeters (https://www.fire.ca.gov/what‐we‐do/fire‐resource‐assessment‐program/fire‐perimeters), to determine the total hectares of fire in giant sequoia groves 1910–2011. The FRAP data base, beginning in 1910, reasonably represents the post‐fire suppression period, given that fire‐scar studies indicate that fire frequency dropped below the historical range of variability in giant sequoia groves circa 1900 (Swetnam et al. 2009, figure 8B). I applied the estimate of historical percent high‐severity fire for giant sequoia groves (8%) from Williams et al. (2023) to the area burned in sequoia groves 1910–2011, adding this area to the high‐severity fire area for 2012–2024 to derive a high‐severity fire area total for 1910–2024 in giant sequoia groves. I calculated the high‐severity fire rotation interval as the period of observation (115 years) divided by the fraction of the study area burned at high severity during that period (Baker 2014). I followed the same procedure described above to determine the post‐fire suppression rotation interval for lower‐severity fire (unchanged, low severity, and moderate severity combined) in giant sequoia groves.

I estimated the pre‐fire suppression (pre‐1910) rotation interval of high‐severity fire from several sources. I used existing estimates from four studies which included historical fire regime estimates that apply to the mixed‐conifer/giant‐sequoia subtype (Stephens et al. 2007; Baker 2014; Hanson and Odion 2016a, 2016b; Williams et al. 2023). Two of these studies reported pre‐fire suppression rotation intervals for fire generally but also included the estimated mean percentage of high‐severity fire that occurred during each fire interval (Stephens et al. 2007; Williams et al. 2023), which allows for a simple calculation of the high‐severity fire rotation interval, as described above. Hanson and Odion (2016a, 2016b) directly reported the pre‐fire suppression high‐severity fire rotation interval. For Baker (2014), I used the high‐severity fire polygons for the 110‐year pre‐fire suppression analysis period in that study and overlaid these polygons on giant sequoia groves to determine the fraction of the grove area that burned at high severity during this time interval. I used these figures to calculate the pre‐fire suppression high‐severity fire rotation interval in giant sequoia groves. For pre‐fire suppression rotation intervals of lower‐severity fire, I used estimates from three sources, Stephens et al. (2007), Swetnam et al. (2009), and Williams et al. (2023). These studies provided direct lower‐severity fire rotation interval estimates for the pre‐fire suppression period.

These sources, pertaining to pre‐fire suppression high‐severity fire rotation intervals, are very similar but not identical in their definitions or descriptions of high‐severity fire. Baker (2014) equated high‐severity fire with 75–100% timber volume mortality, based on a historical source, and estimated that 75% timber volume mortality would be higher than 76% basal area mortality (Baker 2014). Williams et al. (2023) described high‐severity fire as 95%–100% tree mortality. Stephens et al. (2007) equated high‐severity fire with ~100% mortality, and Hanson and Odion (2016a, 2016b) used a historical data set that described high‐severity fire areas as those in which fire had killed all merchantable timber, again ~100% tree mortality. While high‐severity fire in the RAVG database is described as 76%–100% basal area mortality, research has found that 77% of the plots in the RAVG high‐severity fire category have 100% basal area mortality, and an additional 10% have 90%–99% basal area mortality (Miller and Quayle 2015). Accordingly, high‐severity fire is reasonably comparable across sources.

For an additional estimate of the pre‐fire suppression high‐severity fire rotation interval, I estimated the pre‐fire suppression (pre‐1910) rotation interval for mortality of mature giant sequoias using age class distribution data for three giant sequoia groves provided in Stephenson (Stephenson 1994, figure 2). I reasoned that infrequent intervals of high‐severity fire historically functioned as the primary agent of mature giant sequoia mortality and, therefore, the estimate of the pre‐fire suppression rotation interval for mortality of mature giant sequoias would serve as a reasonable proxy for the historical high‐severity fire rotation interval. I made two estimates, using pre‐1900 and pre‐1800 age class data, respectively, from Stephenson (1994) (the youngest age class was 94–193 years old in 1994, and would be 125–224 years old now). For these estimates, I used the negative exponential form of the Weibull distribution, which assumes the probability of mortality from fire does not change with the passage of time since the last fire mortality event (Johnson and Gutsell 1994; Cyr et al. 2009). This assumption has held true in empirical fire history research (Cyr et al. 2009). Under the negative exponential form of the Weibull distribution, the mortality rotation interval is estimated by determining the number of years, back through time before the starting year, at which 63.2% of the age class frequency distribution is younger than the resulting year (Johnson and Gutsell 1994). Higher proportions of the total age class distribution in the younger age classes are associated with shorter rotation intervals and low proportions of the total age class distribution in the younger age classes are associated with long rotation intervals.

### 2.2 Fire Suppression and High‐Severity Fire

Among the recent, large lightning fires was the KNP Complex fire of 2021 (35,737 ha). This wildfire was unusual in that it burned through multiple giant sequoia groves that had no logging history, since the fire occurred on protected National Park lands. In addition, the fire spread through some giant sequoia groves that, unlike most other groves, had some intermittent fire activity over the previous decades. This situation created an opportunity to study the relationship between the time since the previous fire and high‐severity fire occurrence.

I analyzed the null hypothesis that there is no correlation between percent high‐severity fire and time‐since‐fire (fire exclusion) in giant sequoia groves within the KNP Complex fire. I used the following time‐since‐fire categories for this analysis: 1–5, 6–10, 11–15, 16–20, 21–30, 31–50, 51–100, and > 100 years between the previous fire and the KNP Complex fire in 2021. In all giant sequoia grove areas in SEKI that were wholly or partially within the perimeter of the KNP Complex fire, I used the FRAP fire perimeter database and the RAVG fire severity database to determine the percent high‐severity fire in each time‐since‐fire category. I used a Spearman's rank correlation test (Glantz 2005) to analyze whether there is a correlation between the time‐since‐fire categories and percent high‐severity fire.

### 2.3 Lower‐Severity Prescribed Fire and Giant Sequoia Regeneration

I investigated the hypothesis, articulated by land managers, that initial giant sequoia seedling density after lower‐severity prescribed fire would be correlated with higher sequoia regeneration densities in later years, using a SEKI field plot data set (1969–2016) of giant sequoia regeneration after lower‐severity prescribed fires from 1 year to 20 years after prescribed fires. Data for sequoia regeneration surveys up to 5 years after prescribed fire from this data set are provided in Stephenson, Caprio, et al. (2024), who described the prescribed fires at issue as being comprised of low/moderate‐severity fire with some very small high‐severity fire patches, generally fractions of a hectare in size (Stephenson, Soderberg, et al. 2024; Stephenson, Caprio, et al. 2024). I refer to these prescribed fires as having “lower‐severity fire” effects in this study. Stephenson, Caprio, et al. (2024) did not include SEKI prescribed fire and sequoia regeneration data beyond 5 years post‐burn. Data for prescribed fire and sequoia regeneration surveys beyond 5 years post‐burn were provided to me by SEKI and are found in Appendix S1 of this study. Further, an interactive map showing the location of each prescribed fire plot, with coordinates, can be found at: https://ginfo.maps.arcgis.com/apps/mapviewer/index.html?webmap=9c2278f3c59c4813bd4c5b77207bcbff.

For the analyses of correlation between sequoia regeneration density at 1 and 5 years after prescribed fire, and correlation between 1 and 10 years after prescribed fire, I used the same plots from this SEKI data set that were used in Stephenson, Caprio, et al. (2024), except that I used only prescribed fire plots and excluded four plots from this data set that burned in wildfires. I used Spearman's rank correlation tests (Glantz 2005) to determine whether there is a correlation between giant sequoia regeneration density at 1 year post‐burn and 5 years post‐fire, and whether there is a correlation between giant sequoia regeneration density at 1 year post‐burn and 10 years post‐burn. For each analysis, I only used plots for which sequoia regeneration data had been gathered by SEKI for both post‐burn time periods. There were an insufficient number of prescribed fire plots with data at both 1 year post‐burn and 20 years post‐burn to analyze correlation between these two post‐burn time periods.

In addition, I used the SEKI lower‐severity prescribed fire plots to assess the proportion of plots devoid of giant sequoia regeneration (0 seedlings/saplings per plot) at 1, 5, 10, and 20 years after lower‐severity prescribed fire. To ensure an even comparison of sequoia regeneration non‐occupancy at each post‐burn time period, I used only 250‐m^2^ plots from the SEKI data set. Also, as in the correlation analysis above, I used only lower‐severity prescribed fire plots and excluded four wildfire plots in this data set. I also excluded plots with an unknown management history (these were generally plots that the SEKI data set indicated may have been managed with thinning and pile burning instead of the application of prescribed fire). I analyzed the null hypothesis that there would be no change in the proportion of non‐occupancy of sequoia regeneration over time after lower‐severity prescribed fire using a Chi‐square test for trends in binomial proportions (Rosner 2000).

## 3 Results

### 3.1 Rates of High‐Severity Fire Before and After Fire Suppression

I found that, in the 115‐year time period from 1910 through 2024, fire affected 82.0% of the 10,360 ha of total sequoia grove area (Figure 2), and 16.9% of the total grove area experienced high‐severity fire, for a high‐severity fire rotation interval of 680 years (Table 1, Appendix S2). Pre‐fire suppression high‐severity fire rotation interval estimates are shorter, that is, corresponding to more frequent high‐severity fire prior to fire suppression, with estimates of high‐severity fire rotation intervals ranging from 232 years to 473 years (Table 1, Appendixes S2 and S3). The pre‐fire suppression high‐severity fire rotation interval from Baker (2014) was 300 years (Appendix S3), and Hanson and Odion (2016a, 2016b) reported a pre‐fire suppression high‐severity fire rotation interval of 273 years. Williams et al. (2023) reported 8% high‐severity fire on average every 31 years, for a pre‐fire suppression rotation interval of 387 years. Stephens et al. (2007) reported 5% high‐severity fire on average every 20 years, for a pre‐fire suppression high‐severity fire rotation interval of 400 years. The pre‐fire suppression rotation interval for mortality of mature giant sequoias was 232 years for pre‐1900 giant sequoias and 473 years for pre‐1800 giant sequoias.

**TABLE 1:** Post‐fire suppression and pre‐fire suppression estimates of high‐severity fire (HS) rotation intervals and percent high‐severity fire per century.

Sources and time periods	HS rotation interval (years)	% HS per century
Post‐suppression, 1910–2024	680	14.7
Pre‐suppression: Baker 2014	300	33.3
Pre‐suppression: Hanson and Odion 2016a, 2016b	273	36.6
Pre‐suppression: Williams et al. 2023	387	25.8
Pre‐suppression: Stephens et al. 2007	400	25.0
Pre‐suppression: age‐class analysis, pre‐1900	232	43.1
Pre‐suppression: age‐class analysis, pre‐1800	473	21.1

I also found that, in the 115‐year post‐fire suppression time period, the rotation interval for lower‐severity fire was 177 years (Appendix S2). The pre‐fire suppression rotation interval estimates for lower‐severity fire are much shorter, indicating more lower‐severity fire prior to fire suppression. Estimates in existing scientific literature of pre‐fire suppression rotation intervals for lower‐severity fire are 31 years (Williams et al. 2023), 20 years (Stephens et al. 2007), and 16 years (Swetnam et al. 2009).

### 3.2 Fire Suppression and High‐Severity Fire

There was no significant correlation between time‐since‐fire (TSF) and percent high‐severity fire, and the null hypothesis was not rejected (*r*_s_ = 0.238, *p* = 0.570, Table 2, Appendix S4).

**TABLE 2:** Time‐since‐fire (TSF) and percent high‐severity fire in giant sequoia groves within the KNP Complex fire.

TSF (years)	% high severity	Hectares
1–5	8.9	185
6–10	10.1	335
11–15	20.6	232
16–20	8.1	180
21–30	21.3	93
31–50	64.1	101
51–100	6.2	65
> 100	21.1	567

### 3.3 Lower‐Severity Prescribed Fire and Giant Sequoia Regeneration

**FIGURE 3:**
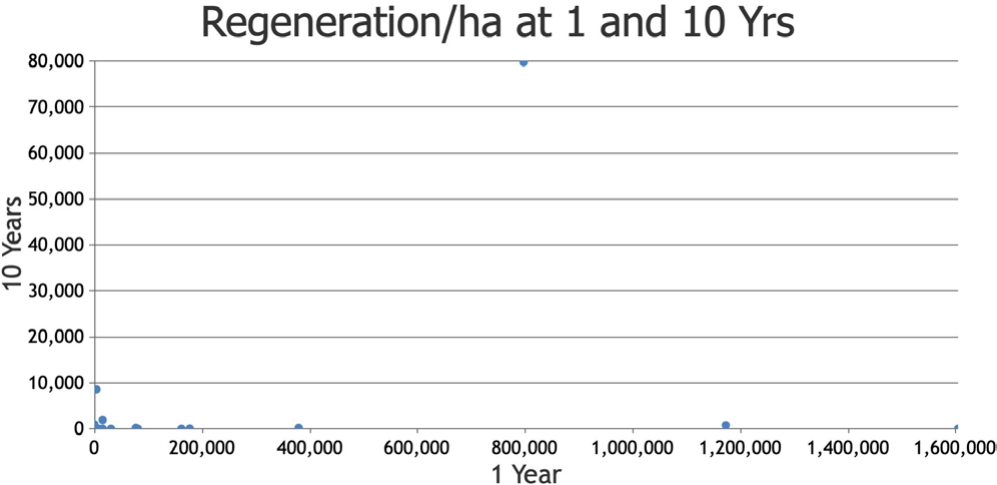
Giant sequoia regeneration density (stems/ha) at 1 and 10 years following lower‐severity prescribed fire in the same plots.

There was a significant correlation between giant sequoia regeneration density (seedlings/ha) at 1 year after prescribed fire and sequoia regeneration density at 5 years (*r*_s_ = 0.590, *p* = 0.002) post‐burn (Appendix S5). There was not, however, a correlation between sequoia regeneration density at 1 year after lower‐severity prescribed fire and density at 10 years after prescribed fire (*r*_s_ = 0.218, *p* = 0.306, Figure 3, Appendix S5).

**FIGURE 4:**
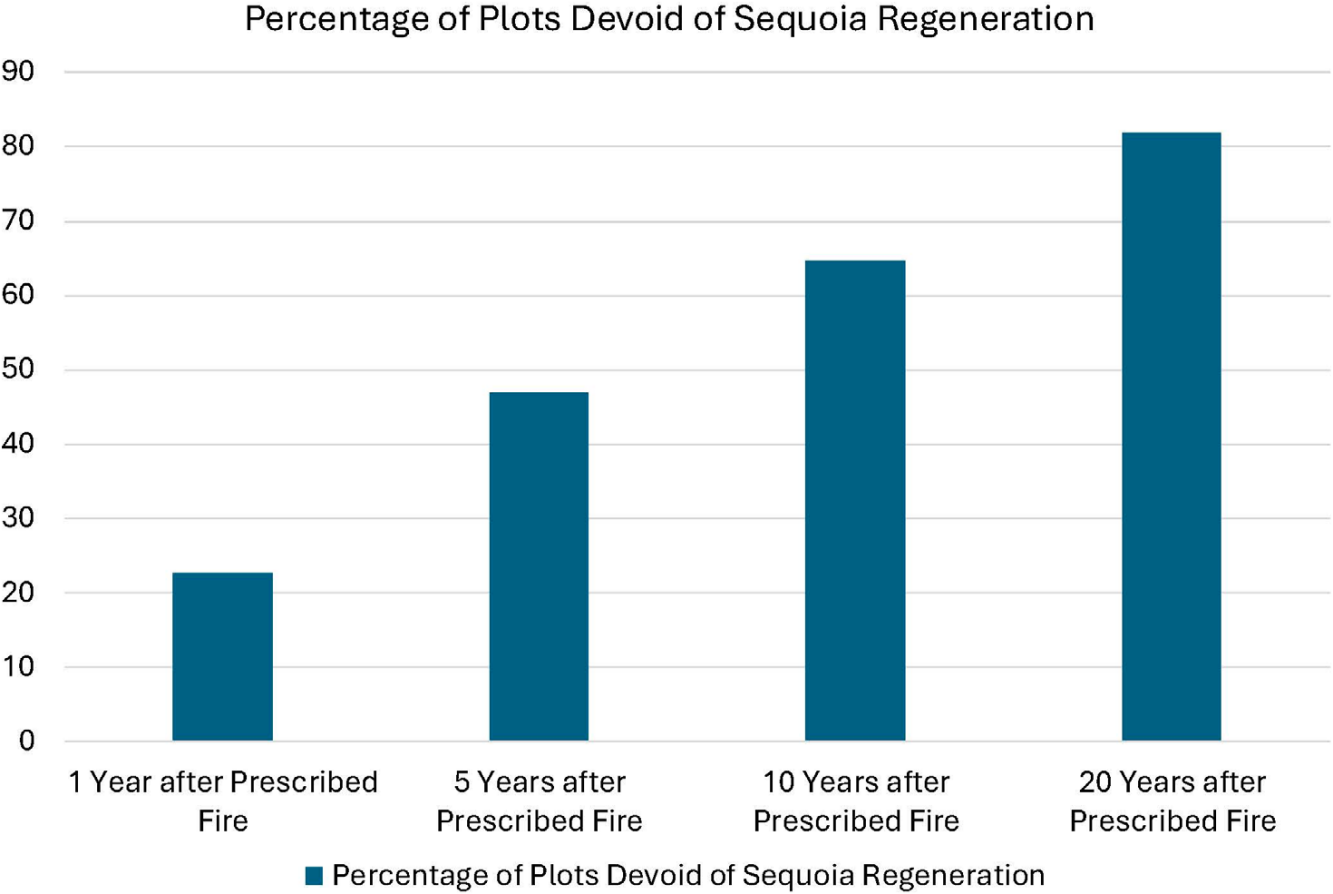
Percentage of Sequoia and Kings Canyon National Parks 250 m^2^ plots devoid of any giant sequoia regeneration at 1, 5, 10, and 20 years after lower‐severity prescribed fire.

Non‐occupancy of giant sequoia regeneration (the proportion of plots devoid of any giant sequoia regeneration) significantly and progressively increased from year 1 (*n* = 31), to year 5 (*n* = 32), to year 10 (*n* = 34), and to year 20 (*n* = 11) after lower‐severity prescribed fire (*χ* ^2^ = 8.16, df = 1, *p* = 0.004, Figure 4, Appendix S6). The null hypothesis was rejected. Plots with no sequoia regeneration increased from 22.6% at 1 year after prescribed fire to 81.8% by 20 years after prescribed fire. At 20 years after lower‐severity prescribed fire, mean giant sequoia regeneration density was 10.9/ha and median density was 0.0/ha (Appendix S6).

## 4 Discussion

Despite the large wildfires that have occurred in giant sequoia groves over the past decade, I found that fire of all severities remains below levels that occurred prior to the beginning of fire suppression policies in 1910. I also found that lower‐severity prescribed fires did not effectively facilitate giant sequoia regeneration over time, unlike findings in coast redwood ( *Sequoia sempervirens* ) forests, where prescribed fire has been successful at facilitating regeneration of redwood seedlings and saplings (Cowman and Russell 2021; Biblin et al. 2025).

In addition, I found no relationship between the years since the previous fire and the percentage of high‐severity fire, contrary to the common hypothesis that, due to fire suppression, long‐unburned forests will have much greater levels of high‐severity fire due to fuel accumulation and increased stand density. While some modeled analyses have reported a weak or equivocal increase in fire severity with increasing decades since the last fire (Steel et al. 2015), most research has either found no relationship between time‐since‐fire and fire severity (Odion and Hanson 2008; van Wagtendonk et al. 2012), or lower levels of high‐severity fire in the most long‐unburned forests (Odion et al. 2004, 2010; Miller et al. 2012). What accounts for this seemingly counter‐intuitive finding? A growing body of science is finding that wildfire behavior is driven mainly by climate and weather factors, especially hot, dry, windy conditions during drought years (Lydersen et al. 2014; Bradley et al. 2016; Reilly et al. 2022), and that denser forests with higher biomass and canopy cover have a cooler, more humid, shadier, and less windy microclimate that is often associated with lower wildfire severity (Zald and Dunn 2018; Lesmeister et al. 2019, 2021; Meigs et al. 2020).

Two recent review articles, Stephenson, Soderberg, et al. (2024) and Keeley and Pausas (2025), hypothesized that, before fire suppression, high‐severity fire may have been largely limited to very small patches typically ~0.1 ha and up to a few hectares (Stephenson, Soderberg, et al. 2024), and suggested that larger patches may have been rare. Stephenson, Soderberg, et al. (2024) based this view on age‐diameter data from Huntington (1914). Stephenson, Soderberg, et al. (2024) stated these data indicated that giant sequoias 3.2 m in diameter at breast height (dbh) had a mean age of 1000 years, and suggested that the number of sequoias of this size and larger in Redwood Mountain Grove implies the absence of larger high‐severity fire patches for at least 1000 years. However, Stephenson, Soderberg, et al. (2024) mis‐cited Huntington (1914), which presented data on diameter *inside bark* at the stump height from 19th‐century logging, a height that was higher than breast height (Stephenson and Demetry 1995). Therefore, Stephenson, Soderberg, et al. (2024) did not account for the famously thick bark of large giant sequoias or the pronounced taper near the base of the trees (Sillett et al. 2015, 2019), leading the authors to underestimate diameter at breast height and overstate tree age. Data on actual dbh‐age relationships indicate that giant sequoias generally reach ~3.2 m dbh by 400 to 600 years of age (Sillett et al. 2015, 2019). Keeley and Pausas (2025) based their assumption about historical high‐severity fire patches upon the proposition that giant sequoia reproduction was “almost completely absent” in larger high‐severity fire patches in 2020–2021 wildfires, citing Soderberg et al. (2024) for this assertion. However, Soderberg et al. (2024) reported giant sequoia seedling densities of ~5000‐15,000/ha even at the upper end of the high‐severity fire spectrum, in crown fire areas, at one year post‐fire (Soderberg et al. 2024, figure 4C). In the supplemental spreadsheet data provided by Soderberg et al. (2024) for their surveys at two years post‐fire, giant sequoia seedling densities in crown fire areas (RdNBR values > 800) were even higher, ~8000‐16,000/ha. Moreover, large high‐severity fire patches dozens or hundreds of hectares in size, and sometimes larger, have been extensively documented in pre‐fire suppression mixed‐conifer forests of the southern and central Sierra Nevada in other research, based on historical U.S. government forest surveys (Baker 2014; Hanson and Odion 2016a, 2016b). The Baker (2014) mapping of large, historical high‐severity fire patches, based on U.S. General Land Office forest surveys conducted in the mid/late‐1800s, had a high degree of accuracy when compared to direct mapping of large high‐severity fire patches in the 19th century by U.S. Geological Survey field crews in the late 1800s (Baker 2014).

The assumption that fire suppression and associated increases in forest density and live biomass has caused unprecedented fire severity in giant sequoia groves (Shive et al. 2022; Meyer et al. 2024; Soderberg et al. 2024; Stephenson, Soderberg, et al. 2024; Stephenson, Caprio, et al. 2024; Keeley and Pausas 2025) has precipitated a series of forest management activities in sequoia groves by land management agencies, including mechanical thinning, post‐fire logging, and tree plantation projects, in the name of wildfire management and reforestation (USDA 2022; USDOI 2023). The rationale for this management approach is that human management, fire suppression, caused a harmful and unnatural outcome and, therefore, human management will solve the perceived problem (USDA 2022, USDOI 2023). In addition to my findings in this study, there are reasons for concern about this approach, including a large body of science finding that mechanical thinning and post‐fire logging are not effective in curbing fire severity (Chen et al. 1999; Donato et al. 2006; Thompson et al. 2007; Hanson 2021, Baker and Hanson 2022), and may tend to kill most of the natural post‐fire sequoia and other conifer regeneration (Donato et al. 2006; Hanson, Chi, Baker, et al. 2024).

Giant sequoia reproduction has often been found to be greatest in high‐severity fire patches (Meyer and Safford 2011; York et al. 2013; Hanson, Chi, Khosla, et al. 2024; Hanson, Chi, Baker, et al. 2024), and high‐severity fire is associated with high, but not complete, levels of mortality of giant sequoias (Shive et al. 2022). For giant sequoia conservation, we do not want too much wildfire, or too little. Monitoring of fire trends in giant sequoia groves in the coming years and decades will be important to inform wildfire policies. When fire is still in deficit, as it is now, land managers should prioritize increasing managed wildfire, prescribed fire, and Indigenous cultural burning (North et al. 2015; Baker et al. 2023). Such burning provides better ecological outcomes and is far less expensive per unit of area than mechanical operations (North et al. 2015), and research demonstrates that there is no need to remove trees prior to burning, even in fire season (during mild fire weather) and in the densest and most long‐unburned forests (Stephens and Finney 2002; Knapp et al. 2005).

Yet my findings do not suggest that lower‐severity prescribed fire should be deployed by land managers as a surrogate for mixed‐severity wildfires, or as a tactic to prevent and suppress wildfires. I found no relationship between initial density of sequoia seedlings and sequoia regeneration density at 10 years post‐burn in prescribed fire areas, and 82% of the plots were devoid of any giant sequoia regeneration 20 years after lower‐severity prescribed fire, contrary to assumptions that prescribed fires will tend to result in substantial and enduring sequoia regeneration and succession to maturity, facilitating a stable or increasing population (York et al. 2013; Soderberg et al. 2024; Stephenson, Soderberg, et al. 2024; Stephenson, Caprio, et al. 2024; Keeley and Pausas 2025). Prescribed fires have a place and a role in giant sequoia conservation, especially at times or in areas where lower‐severity fire is substantially in deficit (Baker et al. 2023). Nevertheless, the body of existing evidence suggests that we might consider accepting mixed‐severity wildfires as an ecologically vital, and evolutionarily ancient (He et al. 2016; Lamont et al. 2019, 2020), process for giant sequoias and other serotinous conifers (Schoennagel et al. 2003; Briand et al. 2015; Su et al. 2015; Ladd et al. 2022; Pelletier and de Lafontaine 2023; Liu et al. 2025), one that is intrinsic to giant sequoia conservation.

## Author Contributions

**Chad T. Hanson:** conceptualization (lead), data curation (lead), formal analysis (lead), funding acquisition (lead), investigation (lead), methodology (lead), project administration (lead), resources (lead), software (lead), supervision (lead), validation (lead), visualization (lead), writing – original draft (lead), writing – review and editing (lead).

## Funding

This research was funded by Environment Now, grant #2025.

## Conflicts of Interest

The author declares no conflicts of interest.

## Supporting information


**Appendix S1:** ece373286‐sup‐0001‐AppendixS1.pdf.

**Appendix S2:** ece373286‐sup‐0002‐AppendixS2.xlsx.

**Appendix S3:** ece373286‐sup‐0003‐AppendixS3.xlsx.

**Appendix S4:** ece373286‐sup‐0004‐AppendixS4.xlsx.

**Appendix S5:** ece373286‐sup‐0005‐AppendixS5.xlsx.

**Appendix S6:** ece373286‐sup‐0006‐AppendixS6.xlsx.

## Data Availability

The raw data for this study, including coordinates, can be found in Appendixes S1–, S6.
