# Supplementary material for: Is Wildfire Suppression in Giant Sequoia Groves a Problem, a Solution, or Neither?
Source: Ecol Evol. 2026 Mar 16;16(3):e73286. doi: 10.1002/ece3.73286 (PMC13093354; doi:10.1002/ece3.73286)
Supplement: Supplementary file 1 — Appendix S1: ece373286‐sup‐0001‐AppendixS1.pdf. [file ECE3-16-e73286-s003.pdf]

SEKI Giant Sequoia FMH Plot Seedling Data (Nov. 15, 2024)

Attached is a PDF document with post-fire exclusion burn seedling data from SEKI FMH plots as well as seedling data from unburned FMH plots (most of the latter plots were eventually burned as burn units shifted).

Values given are raw field numbers from the FMH database for the plot area that was sampled. The values have not undergone a strict quality check (verifying database values with original paper data sheets), which would be done prior any publication.

Sequoia seedling data collected in FMH plots (burned or unburned) is done pre-fire, at one-year post-fire, at two years (but recently discontinued), five years, 10-years, and at 20-years. There are a few sample dates in off years that are also included, which occurred for a variety of reasons. Data already provided in Stephenson et al. 2024 are not included (pre-burn, first-year post-fire, second-year post-burn, and five-years post-burn). There are also data included from several plots not used in the Stevenson et al. 2023 analysis (see comments) because they had an uncertain treatment history (for example, they may have undergone a thinning treatment prior to being treated with fire). Some plots sampled at 10 years were not resampled at 20 years.

Data provided include:

1) Plot designation
2) Monitoring status (burned or unburned) and years since burn
3) Date sampled
4) Live/Dead seedling status
5) Seedling count
6) User variable indicating seedling
7) Area sampled (square meters)
8) Species sampled (all giant sequoias)
9) Comments

MacroPlot Name	Monitoring Status	Sample Date	Status L/D	Count	UV3	Area_m^2	Species	Visited	Comment
Burned (01)									
B:FCADE1T09:063	Re01Year10	8/7/02	L	1993	seed	250	SEGI1	TRUE	
B:FCADE1T09:063	Re01Year10	8/7/02	D	4	seed	250	SEGI1	TRUE	
B:FSEGI1T08:006	Re01Year10	9/26/92		0		250	SEGI1	TRUE	Unknown treatment history (thinning, pile burning?) & not used for analysis
B:FSEGI1T08:007	Re01Year10	7/21/92		0		250	SEGI1	TRUE	Unknown treatment history (thinning, pile burning?) & not used for analysis
B:FSEGI1T08:011	Re01Year10	6/23/94		0		250	SEGI1	TRUE	
B:FSEGI1T08:012	Re01Year10	6/30/94	L	214	seed	250	SEGI1	TRUE	
B:FSEGI1T08:015	Re01Year10	7/10/08		0		250	SEGI1	TRUE	Unknown treatment history (thinning, pile burning?) & not used for analysis
B:FSEGI1T08:022	Re01Year10	8/23/95		0		250	SEGI1	TRUE	
B:FSEGI1T08:023	Re01Year10	8/23/95		0		250	SEGI1	TRUE	
B:FSEGI1T08:024	Re01Year10	7/7/97	L	7	seed	250	SEGI1	TRUE	
B:FSEGI1T08:030	Re01Year10	7/30/96		0		250	SEGI1	TRUE	
B:FSEGI1T08:032	Re01Year10	9/4/96		0		250	SEGI1	TRUE	
B:FSEGI1T08:042	Re01Year10	7/1/97		0		250	SEGI1	TRUE	
B:FSEGI1T08:043	Re01Year10	7/18/97		0		250	SEGI1	TRUE	
B:FSEGI1T08:050	Re01Year10	10/22/97	L	353	seed	250	SEGI1	TRUE	
B:FSEGI1T08:050	Re01Year10	10/22/97	D	3	seed	250	SEGI1	TRUE	
B:FSEGI1T08:051	Re01Year10	10/21/97		0		250	SEGI1	TRUE	
B:FSEGI1T08:067	Re01Year10	8/8/12	L	1	seed	250	SEGI1	TRUE	Unknown treatment history (thinning, pile burning?) & not used for analysis
B:FSEGI1T08:068	Re01Year10	6/30/00		0		250	SEGI1	TRUE	
B:FSEGI1T08:069	Re01Year10	7/21/00		0		250	SEGI1	TRUE	
B:FSEGI1T08:070	Re01Year10	8/1/02	L	1	seed	250	SEGI1	TRUE	
B:FSEGI1T08:071	Re01Year10	7/24/02	L	19	seed	250	SEGI1	TRUE	
B:FSEGI1T08:072	Re01Year10	8/6/02	L	5	seed	250	SEGI1	TRUE	
B:FSEGI1T08:073	Re01Year10	8/5/02	L	5	seed	250	SEGI1	TRUE	
B:FSEGI1T08:074	Re01Year10	8/4/00		0		250	SEGI1	TRUE	
B:FSEGI1T08:075	Re01Year10	8/6/02		0		250	SEGI1	TRUE	
B:FSEGI1T08:079	Re01Year10	9/8/00		0		250	SEGI1	TRUE	
B:FSEGI1T08:080	Re01Year10	9/7/00		0		250	SEGI1	TRUE	
B:FSEGI1T08:081	Re01Year10	6/17/01	L	5	seed	250	SEGI1	TRUE	
B:FSEGI1T08:082	Re01Year10	8/15/01		0		250	SEGI1	TRUE	
B:FSEGI1T08:087	Re01Year10	8/24/01	L	40	seed	250	SEGI1	TRUE	
B:FSEGI1T08:088	Re01Year10	8/23/01	L	288	seed	250	SEGI1	TRUE	

B:FSEGI1T08:093	Re01Year10	8/11/05	L	1	seed	250	SEGI1	TRUE	
B:FSEGI1T08:095	Re01Year10	8/11/05		0		250	SEGI1	TRUE	
B:FSEGI1T08:106	Re01Year10	6/15/09	L	7	seed	250	SEGI1	TRUE	
B:FSEGX1T08:001	Re01Year10	6/11/92		0		250	SEGI1	TRUE	
B:FSEGX1T08:002	Re01Year10	6/25/92	L	24	seed	250	SEGI1	TRUE	
B:FSEGX1T08:003	Re01Year10	7/10/92	L	47	seed	250	SEGI1	TRUE	
B:FSEGX1T08:004	Re01Year10	6/11/92		0		250	SEGI1	TRUE	
B:FSEGI1T08:007	Re01Year20	8/26/02		0		250	SEGI1	TRUE	Unknown treatment history (thinning, pile burning?) & not used for analysis
B:FSEGI1T08:006	Re01Year20	8/28/02		0		250	SEGI1	TRUE	Unknown treatment history (thinning, pile burning?) & not used for analysis
B:FSEGI1T08:022	Re01Year20	8/29/05		0		250	SEGI1	TRUE	
B:FSEGI1T08:030	Re01Year20	8/10/06		0		250	SEGI1	TRUE	
B:FSEGI1T08:032	Re01Year20	8/21/06		0		250	SEGI1	TRUE	
B:FSEGI1T08:050	Re01Year20	7/30/07	L	231	seed	250	SEGI1	TRUE	
B:FSEGI1T08:050	Re01Year20	7/30/07	D	2	seed	250	SEGI1	TRUE	
B:FSEGI1T08:051	Re01Year20	7/30/07		0		250	SEGI1	TRUE	
B:FSEGI1T08:088	Re01Year20	8/23/11	L	5	seed	250	SEGI1	TRUE	
B:FSEGI1T08:088	Re01Year20	8/23/11	D	1	seed	250	SEGI1	TRUE	
B:FSEGI1T08:087	Re01Year20	8/24/11	L	50	seed	250	SEGI1	TRUE	
B:FSEGI1T08:081	Re01Year20	10/3/11	L	2	seed	250	SEGI1	TRUE	
B:FSEGI1T08:071	Re01Year20	8/22/12	L	3	seed	24	SEGI1	TRUE	
B:FSEGI1T08:070	Re01Year20	8/29/12	L	1	seed	250	SEGI1	TRUE	
							SEGI1		
B:FSEGI1T08:022	Re01Year03	7/6/88	L	20	seed	250	SEGI1	TRUE	
B:FSEGI1T08:023	Re01Year03	7/7/88		0		250	SEGI1	TRUE	
B:FSEGX1T08:004	Re01Year04	8/25/86	L	17	seed	250	SEGI1	TRUE	
B:FSEGI1T08:007	Re01year28	7/20/10		0		250	SEGI1	TRUE	Unknown treatment history (thinning, pile burning?) & not used for analysis
B:FSEGI1T08:052	Re01year09	7/16/97		0		250	SEGI1	TRUE	
B:FSEGI1T08:068	Re01year13	7/10/03		0		250	SEGI1	TRUE	
B:FSEGI1T08:043	Re01year16	7/8/03		0		250	SEGI1	TRUE	
B:FSEGI1T08:024	Re01year11	8/7/98	L	5	seed	250	SEGI1	TRUE	
B:FSEGI1T08:006	Re01year24	6/13/06		0		250	SEGI1	TRUE	Unknown treatment history (thinning, pile burning?) & not used for analysis
B:FSEGI1T08:012	Re01year12	7/15/96	L	63	seed	250	SEGI1	TRUE	
B:FSEGI1T08:011	Re01year12	7/16/96	L	2	seed	250	SEGI1	TRUE	

UnBurned (00)

C:FSEGI1T08:015	00Pre	7/1/83		0		250	SEGI1	TRUE	Unknown treatment history (thinning, pile burning?) & not used for analysis
C:FSEGI1T08:031	00Pre	7/31/86		0		250	SEGI1	TRUE	
B:FSEGI1T08:067	00Pre	7/7/90	D	2	seed	250	SEGI1	TRUE	Unknown treatment history (thinning, pile burning?) & not used for analysis
C:FSEGI1T08:005	00Pre	8/24/82		0		250	SEGI1	TRUE	
C:FSEGI1T08:008	00Pre	8/16/82		0		250	SEGI1	TRUE	
B:FSEGI1T08:085	00Pre	10/3/91		0		250	SEGI1	TRUE	
C:FSEGI1T08:111	00Pre	8/13/07		0		250	SEGI1	TRUE	
C:FSEGI1T08:033	00Pre	9/6/86		0		250	SEGI1	TRUE	
C:FSEGI1T08:048	00Pre	9/30/87		0		250	SEGI1	TRUE	
C:FSEGI1T08:112	00Pre	8/14/07		0		250	SEGI1	TRUE	
B:FSEGI1T08:086	00Pre	10/3/91		0		250	SEGI1	TRUE	
C:FSEGI1T08:027	00Pre	7/10/86		0		250	SEGI1	TRUE	
C:FSEGI1T08:113	00Pre	8/22/07		0		250	SEGI1	TRUE	
C:FSEGI1T08:111	Re00Year01	7/30/08		0		250	SEGI1	TRUE	
C:FSEGI1T08:112	Re00Year01	7/30/08		0		250	SEGI1	TRUE	
C:FSEGI1T08:113	Re00Year01	8/6/08		0		250	SEGI1	TRUE	
C:FSEGI1T08:015	Re00Year02	8/11/86		0		250	SEGI1	TRUE	Unknown treatment history (thinning, pile burning?) & not used for analysis
C:FSEGI1T08:031	Re00Year02	8/11/88		0		250	SEGI1	TRUE	
C:FSEGI1T08:008	Re00Year02	10/31/83		0		250	SEGI1	TRUE	
B:FSEGI1T08:085	Re00Year02	9/27/93		0		250	SEGI1	TRUE	
C:FSEGI1T08:111	Re00Year02	7/15/09		0		250	SEGI1	TRUE	
C:FSEGI1T08:033	Re00Year02	10/24/88		0		250	SEGI1	TRUE	
C:FSEGI1T08:048	Re00Year02	7/30/89		0		250	SEGI1	TRUE	
C:FSEGI1T08:112	Re00Year02	7/16/09		0		250	SEGI1	TRUE	
B:FSEGI1T08:086	Re00Year02	7/6/93		0		250	SEGI1	TRUE	
C:FSEGI1T08:027	Re00Year02	6/28/88		0		250	SEGI1	TRUE	
C:FSEGI1T08:113	Re00Year02	7/14/09		0		250	SEGI1	TRUE	
C:FSEGI1T08:048	Re00Year04	8/26/91		0		250	SEGI1	TRUE	
C:FSEGI1T08:015	Re00Year05	8/2/88		0		250	SEGI1	TRUE	Unknown treatment history (thinning, pile burning?) & not used for analysis
C:FSEGI1T08:031	Re00Year05	7/16/91		0		250	SEGI1	TRUE	
B:FSEGI1T08:067	Re00Year05	6/30/95	L	1	seed	250	SEGI1	TRUE	Unknown treatment history (thinning, pile burning?) & not used for analysis
C:FSEGI1T08:005	Re00Year05	10/27/87		0		250	SEGI1	TRUE	
C:FSEGI1T08:008	Re00Year05	10/26/87		0		250	SEGI1	TRUE	

B:FSEGI1T08:085	Re00Year05	9/16/96	0	250	SEGI1	TRUE	
C:FSEGI1T08:111	Re00Year05	9/27/12	0	250	SEGI1	TRUE	
C:FSEGI1T08:048	Re00Year05	7/23/92	0	250	SEGI1	TRUE	
B:FSEGI1T08:006	Re00Year05	10/26/87	0	250	SEGI1	TRUE	Unknown treatment history (thinning, pile burning?) & not used for analysis
C:FSEGI1T08:112	Re00Year05	9/20/12	0	250	SEGI1	TRUE	
B:FSEGI1T08:086	Re00Year05	9/16/96	0	250	SEGI1	TRUE	
C:FSEGI1T08:027	Re00Year05	7/13/91	0	250	SEGI1	TRUE	
C:FSEGI1T08:113	Re00Year05	9/20/12	0	250	SEGI1	TRUE	
C:FSEGI1T08:015	Re00Year10	7/14/93	0	250	SEGI1	TRUE	Unknown treatment history (thinning, pile burning?) & not used for analysis
C:FSEGI1T08:031	Re00Year10	8/6/96	0	250	SEGI1	TRUE	
C:FSEGI1T08:005	Re00Year10	7/24/92	0	250	SEGI1	TRUE	
C:FSEGI1T08:008	Re00Year10	7/22/92	0	250	SEGI1	TRUE	
B:FSEGI1T08:085	Re00Year10	9/6/01	0	250	SEGI1	TRUE	
C:FSEGI1T08:048	Re00Year10	10/22/97	0	250	SEGI1	TRUE	
B:FSEGI1T08:086	Re00Year10	9/7/01	0	250	SEGI1	TRUE	
C:FSEGI1T08:027	Re00Year10	7/8/96	0	250	SEGI1	TRUE	
C:FSEGI1T08:031	Re00Year20	8/15/06	0	250	SEGI1	TRUE	
C:FSEGI1T08:005	Re00Year20	8/29/02	0	250	SEGI1	TRUE	
C:FSEGI1T08:008	Re00Year20	8/27/02	0	250	SEGI1	TRUE	
C:FSEGI1T08:033	Re00Year20	8/22/06	0	250	SEGI1	TRUE	
C:FSEGI1T08:048	Re00Year20	7/30/07	0	250	SEGI1	TRUE	
C:FSEGI1T08:027	Re00Year20	8/7/06	0	250	SEGI1	TRUE	
